# O058: Delivering a central line-associated bacteraemia quality improvement programme in three intensive care units, Auckland City and Star Ship Children’s Hospitals, Auckland, New Zealand

**DOI:** 10.1186/2047-2994-2-S1-O58

**Published:** 2013-06-20

**Authors:** S Roberts

**Affiliations:** 1 Department of Microbiology, Auckland District Health Board, Auckland, New Zealand

## Introduction

In 2011 the New Zealand Health Quality & Safety Commission implemented three national quality improvement programmes: Hand Hygiene New Zealand, a surgical site infection surveillance and improvement programme and Target CLAB Zero. The Target CLAB Zero programme, based on the use ‘bundles of care’ for central line (CVL) placement and maintenance was delivered across all 20 District Health Boards (DHB). The aim was to reduce the rate of CVL-associated bacteremia (CLAB) to < 2 /1000 line days.

## Methods

The Target CLAB Zero programme, lead by Counties Manukau DHB and Ko Awatea, used the Institute of Healthcare Improvement collaborative methodology. At Auckland DHB there are three intensive care units; paediatric ICU (PICU) , adult cardiothoracic and vascular ICU (CICU) and adult medical and surgical ICU (DCCM). A working group with nursing staff from each unit developed a single approach to the delivery of the programme across all three units. This involved education of staff, auditing and feedback of compliance with the ‘bundles’, and monitoring the rate of CLAB/1000 line days. Baseline rates for CLAB/1000 line days were collected prospectively for two units and estimated retrospectively for one.

## Results

As of March 2013, 15 months after starting the collaborative all three units had achieved at some stage >100 days without a CLAB episode and one unit, CICU has not had a CLAB event since February 2012. The overall CLAB rate per quarter (Jan-Dec 2012) across all three units was 4.2, 0, 0.45 and 1.6 CLAB/1000 line days, respectively.

Other improvement activities include the standardization of blood culture collection practices within the units, updating organisation-wide CVL policy and the development of high risk criteria for each unit.

## Conclusion

The Target CLAB Zero programme has resulted in a significant reduction in the number of CLAB/1000 line days among ICU patients. It is now being rolled out in theatres, emergency departments and other areas in the hospital where CVL are placed. Implementation of this programme has lead to a culture of cooperation amongst the three ICU that did not exist previously.

## Disclosure of interest

None declared.

